# Effectiveness of *Rlm7* resistance against *Leptosphaeria maculans* (phoma stem canker) in UK winter oilseed rape cultivars

**DOI:** 10.1111/ppa.12845

**Published:** 2018-03-23

**Authors:** G. K. Mitrousia, Y. J. Huang, A. Qi, S. N. M. Sidique, B. D. L. Fitt

**Affiliations:** ^1^ Centre for Agriculture, Food and Environmental Management University of Hertfordshire Hatfield Hertfordshire AL10 9AB UK; ^2^ Present address: Laboratory for Pest, Disease and Microbial Biotechnology (LAPDiM) School of Food Science and Technology Universiti Malaysia Terengganu Kuala Nerus 21030 Malaysia

**Keywords:** blackleg, *Brassica napus*, deployment of resistance genes, *Leptosphaeria* spp., *R* gene‐mediated resistance, resistance breeding

## Abstract

The *Rlm7* gene in *Brassica napus* is an important source of resistance for control of phoma stem canker on oilseed rape caused by the fungus *Leptosphaeria maculans*. This study shows the first report of *L. maculans* isolates virulent against *Rlm7* in the UK. *Leptosphaeria maculans* isolates virulent against *Rlm7* represented 3% of the pathogen population when cultivars with the *Rlm7* gene represented 5% of the UK oilseed rape area in 2012/13. However, the *Rlm7* gene has been widely used since then, representing >15% of the UK oilseed rape area in 2015/16. Winter oilseed rape field experiments included cultivars with the *Rlm7* gene, with the *Rlm4* gene or without *Rlm* genes and took place at five sites in the UK over four cropping seasons. An increase in phoma leaf spotting severity on *Rlm7* cultivars in successive seasons was observed. Major resistance genes played a role in preventing severe phoma leaf spotting at the beginning of the cropping season and, in addition, quantitative resistance (QR) in the cultivars examined made an important contribution to control of phoma stem canker development at the end of the cropping season. Deployment of the *Rlm7* resistance gene against *L. maculans* in cultivars with QR in combination with sustainable disease management practices will prolong the use of this gene for effective control of phoma stem canker epidemics.

## Introduction

Major resistance genes have been widely used to protect crops against fungal plant diseases and many breeding companies have deployed them in their programmes over the last 100 years (Stuthman *et al*., 2007; Stukenbrock & McDonald, 2008). However, widespread use of a single resistance gene often results in adaptation of pathogen populations, which evolve to become virulent, rendering the specific resistance gene ineffective (‘boom and bust’ cycle) (Vanderplank, 1968). This is especially true for pathogens with airborne spores and sexual reproduction that have a high evolutionary potential (McDonald & Linde, 2002).

One good example of such a pathogen is *Leptosphaeria maculans* on oilseed rape (*Brassica napus*), which has a high evolutionary potential; new strains are produced by mutations at their effector gene (*AvrLm*) loci, rendering the corresponding major host resistance (*Rlm*) genes ineffective (Sprague *et al*., 2006). This pathogen's evolutionary potential is mainly due to its reproductive system and dispersal ability (McDonald & Linde, 2002). *Leptosphaeria maculans*, together with the closely related pathogen *L. biglobosa*, causes the phoma stem canker disease, which is an important problem on oilseed rape worldwide. In the UK, it causes losses to farmers of >£100 million p.a., despite the use of fungicides costing £20 million p.a. (Fitt *et al*., 2006a; Stonard *et al*., 2010). Cultivation of oilseed rape is of vital importance for the UK agricultural industry and benefits both the farming industry, as a break crop in cereal rotations, and the export market for biodiesel and vegetable oil. The cultivated area of oilseed rape in the UK has increased over the last 40 years and personal communication with breeders has suggested that cultivars with the resistance gene *Rlm7* against *L. maculans* have been widely used recently. However, this has not been confirmed.

Strategies for deployment of specific *Rlm* genes in space and time (Johnson, 1984) have been developed in France, Australia and Canada to enable these genes to remain effective during their use over several growing seasons; for example, TerresInovia in France, http://www.terresinovia.fr and http://www.myvar.fr; Grains Research & Development Corporation (GRDC) Blackleg management guide in Australia, https://www.grdc.com.au; and Blackleg management Canola Council in Canada, https://www.canolacouncil.org/canola-encyclopedia/diseases/blackleg/blackleg-management (Pinochet *et al*., 2004; Marcroft *et al*., 2012a). These strategies were developed because severe phoma stem canker epidemics have occurred in field experiments in France (Brun *et al*., 2000; Daverdin *et al*., 2012) and in commercial oilseed rape crops in France, Australia and Canada (Li *et al*., 2003; Rouxel *et al*., 2003; Sprague *et al*., 2006; Liban *et al*., 2016; Zhang *et al*., 2016) when host resistance mediated by *Rlm* genes was rendered ineffective by changes in the pathogen populations. Occurrence of environmental conditions favourable for the disease is likely to accelerate selection for virulent populations due to the greater amount of inoculum available (McDonald & Linde, 2002; Balesdent *et al*., 2015). Combining *Rlm* genes with quantitative resistance (QR) against *L. maculans* has been suggested as a strategy to increase the effectiveness of *Rlm* genes (Brun *et al*., 2010; Zhang *et al*., 2017). *Rlm* gene‐mediated resistance operates at the leaf infection stage to prevent leaf lesion development, whereas QR operates later in the disease cycle to prevent development of severe phoma stem canker (Fitt *et al*., 2006b).

The single effector gene, *AvrLm4‐7*, in *L. maculans* is recognized by two distinct resistance genes in *B. napus*,* Rlm4* and *Rlm7*. Escape from recognition by *Rlm4* is due to a single base mutation that does not alter recognition by *Rlm7* (Parlange *et al*., 2009). By contrast, escape from recognition by *Rlm7* can be associated with several molecular events, such as repeat‐induced point (RIP) mutations or complete deletion of *AvrLm4‐7* (Daverdin *et al*., 2012). Recent findings about the interaction between *AvrLm4‐7* and *AvrLm3*, the effector gene recognized by the *Rlm3* gene in *B. napus*, have shown that *Rlm3*‐mediated resistance is hidden in the presence of the *L. maculans* allele avirulent against *Rlm7*,* AvrLm7* (Plissonneau *et al*., 2016). Extensive use of the major resistance gene *Rlm7*, without rotation in a particular region in France for 3 years, resulted in an increase in the proportion of virulent *L. maculans* strains (Daverdin *et al*., 2012). However, the resistance gene *Rlm7* has been widely deployed in new oilseed rape cultivars across Europe (Winter & Koopmann, 2016) and it has been suggested that it is more durable than other *Rlm* genes that are commercially available (Clarke *et al*., 2011; Balesdent *et al*., 2015).

This paper investigates the effectiveness of the *Rlm7*‐mediated resistance against *L. maculans* in the UK over the period with the cropping seasons 2010/11 to 2013/14. The effectiveness of *Rlm7* was assessed by examining the emergence of *L. maculans* isolates virulent at the corresponding *AvrLm7* locus. Moreover, the ability of *Rlm7*‐mediated resistance to control initiation of epidemics was examined by assessing phoma leaf spotting in autumn/winter. Finally, the effect of *Rlm7* was examined at the phoma stem canker stage of the epidemics, together with any possible effect of QR. Investigation of the effectiveness of a major resistance gene can provide both insight into the pathogen's evolutionary potential at the corresponding *Avr* locus and information to guide strategies for its deployment.

## Materials and methods

### Determining the presence of the *Rlm7* gene in oilseed rape cultivars popular in the UK

In 2010, the Agriculture and Horticulture Development Board (AHDB) began to perform an annual cultivar survey each summer, estimating the total area in the UK planted with each oilseed rape cultivar. Data from these surveys (http://www.ahdb.org.uk) were used to summarize the most popular winter oilseed rape cultivars in the UK for the period with the cropping seasons 2009/10 to 2015/16. The presence of the *Rlm7* gene was determined by INRA Thiverval‐Grignon, France in some of these oilseed rape cultivars popular in the UK. These experiments involved inoculation of cotyledons with specific *L. maculans* isolates and assessment of lesions at 17 days post‐inoculation (dpi) on a 0 (no symptoms) to 6 (large grey‐green lesions with pycnidial production) scale (Balesdent *et al*., 2001). The specific breeding companies communicated information about the presence/absence of the *Rlm7* gene in each of the other 10 cultivars.

### Oilseed rape field experiments

Field experiments were carried out over four cropping seasons (2010/11, 2011/12, 2012/13 and 2013/14) with the cultivars Adriana (*Rlm4 *+* * QR), Bilbao (*Rlm4*), Drakkar (no known *Rlm* gene), Roxet and Excel (both carrying the *Rlm7* gene) at up to 11 sites in England (Bainton, Banbury, Cowlinge, Harpenden, Harper Adams, Horncastle, Morley, Oadby Lodge Farm, Rothwell, Spalding and Stockbridge; Fig. S1). The experimental field plots (1.8 × 15 m) were not sprayed with fungicide in order to assess the resistance of different cultivars against *L. maculans* and were in a randomized block design with three blocks.

### Phenotypes of *L. maculans* phoma leaf spot lesions in oilseed rape field experiments

Detailed assessments were performed to examine the *L. maculans* lesion phenotypes on leaves of the cultivars Drakkar, Excel and Roxet sampled from field experiments at up to 11 sites (Bainton, Banbury, Cowlinge, Harpenden, Harper Adams, Horncastle, Morley, Oadby Lodge Farm, Rothwell, Spalding and Stockbridge; Fig. S1) in the 2011/12 and 2012/13 cropping seasons. In the 2011/12 cropping season, photos of each leaf were taken and lesions on each leaf were identified as caused by *L. maculans*, based on visual observations following pathogen isolation (Fitt *et al*., 2006a). The areas of up to 10 of these lesions per leaf were measured using the software imageJ on leaf samples from 10 sites (all the sites listed except Horncastle where no samples were obtained).

Dark margins were observed around lesions on cultivars with the *Rlm7* gene (Excel and Roxet) in field experiments, whereas large grey lesions without dark margins were observed on the susceptible cultivar Drakkar. The numbers of *L. maculans* leaf lesions with or without dark margins were recorded and the proportions of *L. maculans* leaf lesions observed on *Rlm7* cultivars that were not surrounded by dark margins were assessed at seven (Cowlinge, Harpenden, Harper Adams, Horncastle, Morley, Rothwell and Stockbridge) and eight (Bainton, Banbury, Cowlinge, Harpenden, Horncastle, Morley, Rothwell and Spalding) sites in the winter of the 2011/12 and 2012/13 cropping seasons, respectively.

### Severity of phoma leaf spotting and phoma stem canker

Severity of phoma leaf spotting and phoma stem canker was assessed on samples of the winter oilseed rape cultivars Adriana, Bilbao, Drakkar, Roxet and Excel at five sites (Banbury, Cowlinge, Harpenden, Rothwell and Spalding; Fig. S1) over the 2010/11, 2011/12 and 2012/13 cropping seasons and at three of these sites (Banbury, Cowlinge and Spalding) in the 2013/14 cropping season. No disease data were obtained at Banbury in the 2010/11 and 2011/12 cropping seasons because disease incidence was small.

Phoma leaf spotting severity was assessed on at least 15 plants per plot of each cultivar at each site or on 10 plants per plot from each of the three replicate plots of each cultivar. Phoma leaf spotting severity was assessed using a 0–3 scale (0: no leaf spots; 1: 1–5 leaf spots per plant, 2: 6–10 leaf spots per plant; 3: >10 leaf spots per plant).

Phoma stem canker was assessed in each of the three replicate plots of each cultivar at each site. Ten plants, randomly selected from each of the three replicate plots, were uprooted and collected in June/July before harvest. Each stem was cut at the stem base to assess severity of basal phoma stem canker and at the upper part to assess severity of upper stem lesions. Symptoms were considered to be upper stem lesions if they were observed >10 cm above the root crown and to be basal cankers if they were at the root crown or between the root crown and 10 cm above it. Basal cankers and upper stem lesions were assessed on a 0–6 scale (modified from that of Lô‐Pelzer *et al*., 2009).

### Statistical analysis of field data

Data for phoma leaf spotting severity and stem canker severity at each site (Banbury, Cowlinge, Harpenden, Rothwell and Spalding) were analysed by analysis of variance (ANOVA) using genstat 17th edition statistical software and mean values were compared using a least significant difference (LSD) calculated at a probability level of *P *=* *0.05 (Payne *et al*., 2011). For each of the cropping seasons (2010/11, 2011/12, 2012/13 and 2013/14) and each of the sites, the mean disease scores (*S*; for phoma leaf spotting severity or stem base canker severity or upper stem lesion severity) on each of the five cultivars Adriana, Bilbao, Drakkar, Excel and Roxet (*Sc*_*i*_, where each of the cultivars, *i *=* *1 to 5) were calculated from the scores in each of the three replicate plots (*S*_*ri*_). For each of the cropping seasons (2010/11, 2011/12, 2012/13 and 2013/14), the mean disease severity score (*S*; for phoma leaf spotting, stem base canker or upper stem lesion) at each site (*Ss*_*j*_) (where each of the sites, *j *=* *1 to 5) was calculated as the mean of the disease scores for the five (*n *=* *5) cultivars (*Sc*_*i*_) at each site.

The relative disease severity (RS; for phoma leaf spotting, stem base canker or upper stem lesion) for each cultivar was then calculated as the ratio of the mean disease score for each of the five cultivars (*Sc*_*i*_) divided by the mean disease score at each site (*Ss*_*j*_) and expressed as a percentage. This percentage relative severity was used to study differences between cultivars by regressing the cultivar mean relative severity (*Sc*_*i*_) against the site mean relative severity (*Ss*_*j*_). The equations used to calculate the mean phoma leaf spotting severity or stem base canker and upper stem lesion severity for each cultivar (*Sc*_*i*_) and mean phoma leaf spotting severity at each site (*Ss*_*j*_) are listed in Table S1.

### Detection of virulent allele frequencies in the *L. maculans* populations

A set of cultivars or lines carrying different *Rlm* genes, *Rlm2*,* Rlm3*,* Rlm4* or *Rlm7* (Balesdent *et al*., 2002), was used to determine the frequencies of the corresponding *Avr* alleles in *L. maculans* isolates obtained from the susceptible cultivar Drakkar and from cultivars with the *Rlm7* gene in the 2011/12 and 2012/13 cropping seasons at different sites in the UK. Cotyledons of 14‐day‐old seedlings were point‐inoculated by wounding with a fine needle and placing 10 μL spore suspension (10^7^ spores mL^−1^) over the wounded area. Plants were incubated in a growth chamber at 20 °C with a 12 h photoperiod, with the first 72 h of incubation under high humidity and darkness. The lesion severity was scored 17 to 21 dpi on a 0 (no symptoms) to 6 (large grey‐green lesions with pycnidial production) scale (Balesdent *et al*., 2001) and the phenotype was characterized as resistant (score 1–3) or susceptible (score 4–6). The isolates were characterized as having the corresponding *Avr* allele if they had produced a resistant interaction on the cultivars or lines with the different *Rlm* genes and as lacking the corresponding *Avr* allele if they produced a susceptible interaction.

### Molecular events in the *avrLm7* *L. maculans* isolates

*Leptosphaeria maculans* isolates that showed a virulent phenotype in the cotyledon phenotype test on the *Rlm7* line 01.23.2.1 were used to determine the molecular events at the *AvrLm7* locus. The *AvrLm4‐7* gene was amplified using external and internal primers and PCRs were done according to Daverdin *et al*. (2012).

PCR products of the *L. maculans* isolate from which the locus was amplified with the external primer set were purified using a PCR purification kit (QIAGEN), following the manufacturer's instructions, and they were sequenced at GATC Biotech Ltd. EMBOSS transeq tool was used to translate the nucleotide sequence to an amino acid sequence. Nucleotide and amino acid sequences were compared to those on the reference avirulence allele (*AvrLm4‐7*; GenBank: AM998638.1 and protein_id = CAQ53119.1, respectively) following sequence alignment using clustal v. 2.1.

### Examining the presence of QR and the *Rlm7* gene in Excel and Roxet

As the UK *L. maculans* populations were found to be mostly avirulent against *Rlm7* (presented in this study), any host resistance associated with the stem canker stage could have been masked in the *Rlm7*‐carrying cultivars (Excel and Roxet), and so the two cultivars were assessed for QR in a controlled environment experiment. The cultivars Drakkar (susceptible to *L. maculans*; no known *Rlm* gene) and the doubled haploid (DH) line A30 were used as susceptible controls. The cultivar Adriana (with *Rlm4 *+* * QR) and the DH line C119 were used as controls for QR. The QR of the DH lines used had been studied in previous experimental work in France (Pilet *et al*., 1998; Jestin *et al*., 2011) and in England (Huang *et al*., 2014b). Plants were inoculated at the base of the petiole, close to the stem, of the first and second true leaves using a petiole inoculation method (Huang *et al*., 2014b) with 10 μL of a conidial suspension (10^7^ spores mL^−1^) of the *L. maculans* isolate H Rox 12.2.1 (*avrLm4, avrLm7*) and were incubated at 20 °C for 40 days. External and internal lesion length was measured and stem canker severity was assessed on a 0–6 scale (Huang *et al*., 2014b; modified from the 1–6 scale of Lô‐Pelzer *et al*., 2009). A *t*‐test was done to determine whether the different cultivars were significantly different (*P *<* *0.05 or *P *<* *0.10) from the susceptible controls, line A30 and cultivar Drakkar.

Single pycnidial isolates obtained from phoma leaf spots in the 2011/12 cropping season that were found to be avirulent against *Rlm7* following detection of *Avr* alleles, were used to inoculate cotyledons of Excel and Roxet to confirm the presence of *Rlm7* in these cultivars. Ten plants of each of Excel, Roxet, the susceptible Drakkar (no *Rlm* gene) and the *Rlm7* line of the differential set 01‐23‐2‐1 (Balesdent *et al*., 2002) were point‐inoculated using a 10 μL spore suspension (10^7^ spores mL^−1^). Plants were incubated in a growth chamber at 20 °C with a 12 h photoperiod, with the first 72 h of incubation under high humidity and darkness. Lesion severity was scored 17 dpi on a 0 (no symptoms) to 6 (large grey‐green lesions with pycnidial production) scale (Balesdent *et al*., 2001).

## Results

### Determination of the use of the *Rlm7* gene against *L. maculans* in oilseed rape cultivars in the UK

The first substantial cultivation of oilseed rape cultivars with the *Rlm7* gene (accounting for more than 5% of the surveyed area in each year) in the UK was in 2013, when one cultivar (Cv H; Fig. S2) was grown on 5% of the area. Two cultivars with the *Rlm7* gene were grown in 2014, accounting for 15% of the area (Cv H on 6% and Cv J on 9% of the area). Nine percent and 7% of the surveyed area was grown with one *Rlm7* cultivar (Cv J) in 2015 and 2016, respectively. The presence of the *Rlm7* gene in other cultivars (indicated as ‘Others’ in Fig. S2) that each accounted for less than 5% of the surveyed area each year was not examined because these cultivars were not identified in the survey. Personal communication with breeding companies has indicated that the current use (2016/17) of the *Rlm7* gene in commercial crops in the UK accounts for >20% of the oilseed rape area.

### Phenotype of *L. maculans* phoma leaf spot lesions in oilseed rape field experiments

Lesions observed in the 2011/12 cropping season on cultivars carrying the *Rlm7* gene were generally smaller (0.11 ± 0.03 cm^2^ and 0.14 ± 0.04 cm^2^ on Excel and Roxet, respectively) than the lesions on the susceptible cultivar Drakkar (0.34 ± 0.04 cm^2^; *P *<* *0.01, SED = 0.030). Lesion area of 78 lesions on Drakkar leaves ranged from 0.07 to 1.14 cm^2^, lesion area of 51 lesions on Excel leaves ranged from 0.02 to 0.35 cm^2^, and lesion area of 40 lesions on Roxet leaves ranged from 0.02 to 0.44 cm^2^.

**Figure 1:**
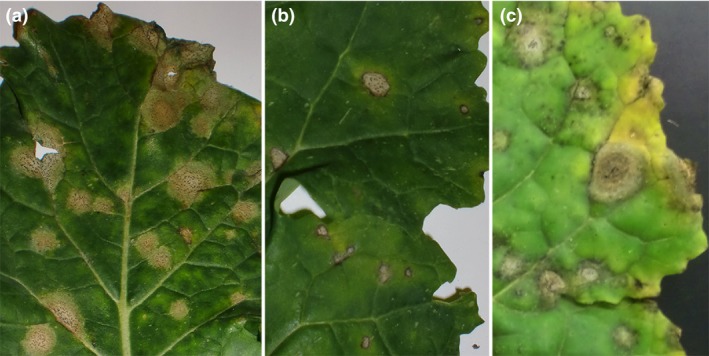
Phoma leaf spot symptoms on leaves of the oilseed rape cultivars Drakkar (no known *Rlm* gene) (a) and Roxet (with the *Rlm7* gene against *Leptosphaeria maculans*) (b, c). Leaves were sampled from field experiments at Cowlinge on 9 December 2011 in the 2011/12 cropping season (a, b) and on 14 January 2013 in the 2012/13 cropping season (c). Phoma leaf spots without (a, c) or with dark margins (b) are illustrated.

**Figure 2:**
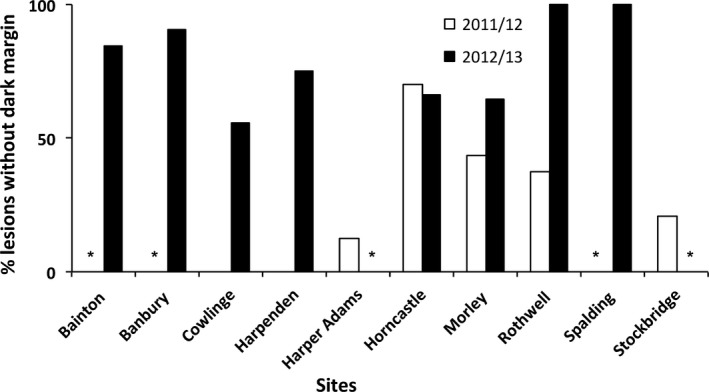
Percentage of phoma leaf spot lesions caused by *Leptosphaeria maculans* that were not surrounded by a dark margin, observed on winter oilseed rape cultivars with *Rlm7* resistance against *L. maculans* at different sites in the UK in the winter of 2011/12 (seven sites) and 2012/13 (eight sites) cropping seasons. An asterisk (*) indicates that there were no samples taken.

All the *L. maculans* leaf lesions observed on *Rlm7* cultivars were surrounded by dark margins at Cowlinge and Harpenden in the winter of the 2011/12 cropping season (Figs 1a & 2). However, 56% of the *L. maculans* leaf lesions at Cowlinge and 75% of those at Harpenden were not surrounded by dark margins in the winter of the 2012/13 cropping season (Figs 1c & 2). An increase in proportion of *L. maculans* leaf lesions without dark margins on *Rlm7* cultivars was also observed at Morley (from 43% to 65%) and at Rothwell (from 38% to 100%) between the 2011/12 and 2012/13 cropping seasons. Eighty‐four percent of the *L. maculans* leaf lesions on *Rlm7* cultivars at Bainton, 91% at Banbury and 100% at Spalding were not surrounded by dark margins in the winter of the 2012/13 cropping season.

### Effects of cropping season, site and cultivar on severity of phoma leaf spot epidemics

Analysis of variance of phoma leaf spotting severity on five cultivars in experimental plots at Banbury, Cowlinge, Harpenden, Rothwell and Spalding in four consecutive cropping seasons (2010/11, 2011/12, 2012/13 and 2013/14) showed that phoma leaf spotting severity differed significantly (*P *<* *0.01) between cropping seasons. There were also significant effects of both cultivar and site on phoma leaf spotting severity (*P *<* *0.01).

**Figure 3:**
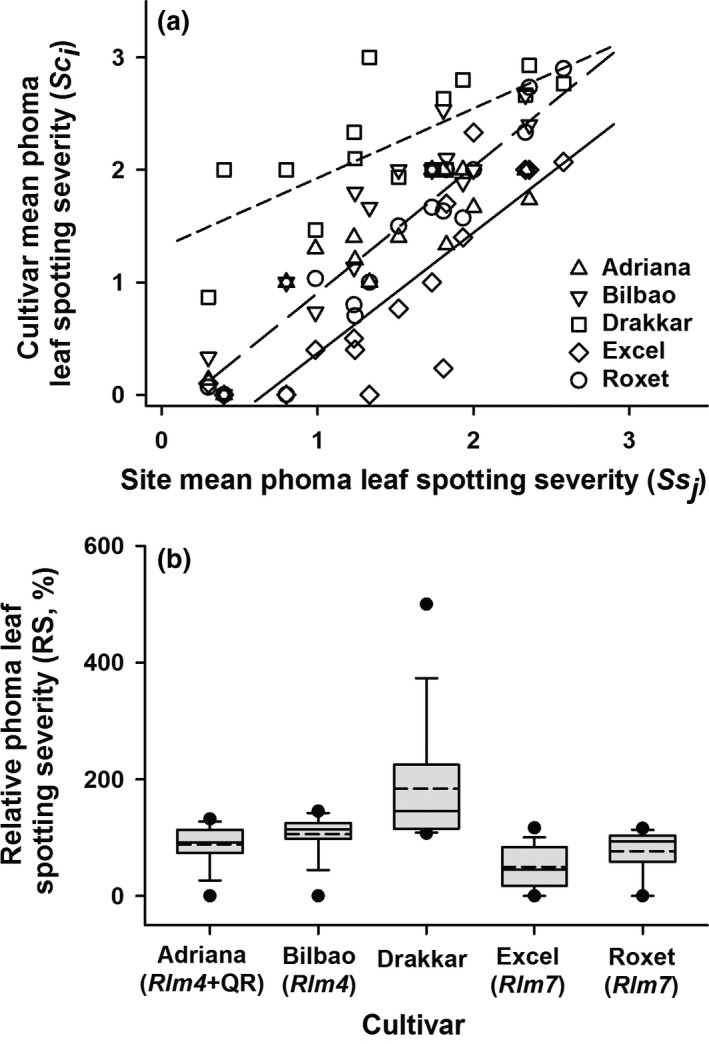
(a) Relationship between mean phoma leaf spotting severity on each cultivar (*Sc*_*i*_) and mean phoma leaf spotting severity at each site (*Ss*_*j*_) over four cropping seasons (2010/11, 2011/12, 2012/13 and 2013/14). Analysis of position and parallelism showed that these data best fitted three non‐parallel linear lines, accounting for 81.1% of the variation (71 d.f.). There was one line (*Sc*_*i*_ **= 0.62*Ss*_*j*_ **+ 1.31) for cultivar Drakkar (– – –), one line (*Sc*_*i*_ **= 1.13 *Ss*_*j*_ **− 0.22) for the cultivars Adriana, Bilbao and Roxet (^_ _ _^) and one line (*Sc*_*i*_ = 1.02 *Ss*_*j*_ *− *0.70) for cultivar Excel (^___^). (b) The distribution in relative phoma leaf spotting severity (RS) for each cultivar. The relative severity was calculated as a ratio and expressed as a percentage $\text{RS}(\%)=\frac{Sc_{i}}{Ss_{j}}\times 100$ , where *Sc*_*i*_ is the mean disease severity for each cultivar *i* (Drakkar, Excel, Roxet, Adriana or Bilbao) and *Ss*_*j*_ is the mean disease severity at each site *j* (Banbury, Cowlinge, Harpenden, Rothwell or Spalding). Each box‐plot shows the mean (– – –) and the median (^___^) percentage RS. The lower and upper boundaries of the boxes indicate the percentage RS for the 25th and 75th percentiles, while whisker bars above and below each box indicate the percentage RS for the 5th and 95th percentiles. Black dots below and above each box‐plot represent the minimum and maximum values, respectively.

The ANOVAs showed that there were significant differences in phoma leaf spotting severity between the five cultivars (*P *<* *0.05). Mean phoma leaf spotting severity for each cultivar was plotted against mean severity at each site (Fig. 3a). The relationship between the mean phoma leaf spotting severity for each cultivar and the mean phoma leaf spotting severity at each site was linear. When an analysis of position and parallelism was done, three distinct groups were identified (Fig. 3a), fitted best by three non‐parallel lines, accounting for 81.1% of the variation (71 d.f.). Thus, most severe phoma leaf spotting symptoms were on Drakkar (fitted by one line: *Sc*_*i*_ = 0.62*Ss*_*j*_ **+ 1.31), there was an intermediate group including Adriana, Bilbao and Roxet (fitted by one line: *Sc*_*i*_ **= 1.13*Ss*_*j*_ – 0.22) and Excel had the least severe phoma leaf spotting symptoms (fitted by one line: *Sc*_*i*_ **= 1.02*Ss*_*j*_ – 0.70; Fig. 3a).

The distribution in the relative phoma leaf spotting severity on each cultivar (Fig. 3b) showed that phoma leaf spotting score was greatest on Drakkar, which was the cultivar on which the greatest variation in relative phoma leaf spotting severity occurred. The second greatest relative cultivar phoma leaf spotting severity score was on Bilbao, Adriana and Roxet and the smallest on Excel.

### Severity of phoma leaf spotting over time

**Figure 4:**
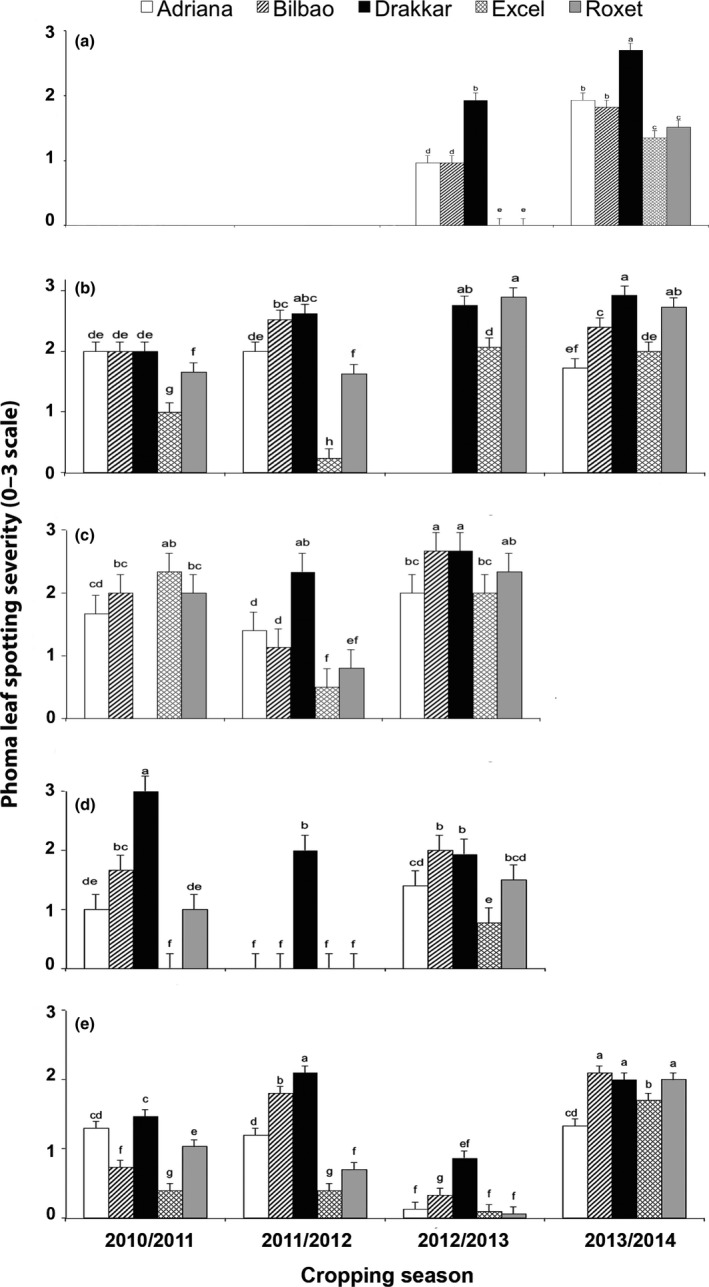
Phoma leaf spot severity (*Leptosphaeria maculans*) on leaves of oilseed rape cultivars Adriana [*Rlm4 *+ quantitative resistance (QR)], Bilbao (*Rlm4*), Drakkar (no known *Rlm* gene), Excel and Roxet (both with *Rlm7*) in established field experiments at Banbury (a), Cowlinge (b), Harpenden (c), Rothwell (d) and Spalding (e) in the 2010/11, 2011/12, 2012/13 and 2013/14 cropping seasons. Phoma leaf spot severity is expressed on a 0–3 scale (0: 0 leaf spots; 1: 1–5 leaf spots per plant, 2: 6–10 leaf spots per plant; 3: >10 leaf spots per plant). The mean severity was calculated from scores on 10 plants from each of the three replicate plots. Mean scores designated by the same letter are not significantly different (*P *>* *0.05).

There was an increase in severity of phoma leaf spotting on *Rlm7* cultivars with time (Fig. 4a–e). No symptoms were observed on Excel and Roxet at Banbury in December 2012 (Fig. 4a). However, phoma leaf spotting (score *c*. 1.5) was observed on the *Rlm7* cultivars in December 2013, with no significant differences between them (*P *<* *0.05; Fig. 4a). While severity of phoma leaf spotting was low on the *Rlm7* cultivars at Cowlinge in December 2010 and in December 2011 (scores *c*. 0.2–1.7), higher scores were observed on Roxet, *c*. 2.8, and on Excel, *c*. 2.1, in January 2013 and December 2013 (Fig. 4b).

Similarly, there was an increase of severity in phoma leaf spotting on *Rlm7* cultivars at Harpenden and Rothwell in the 2012/13 cropping season compared to the previous cropping seasons (Fig. 4c,d). Phoma leaf spotting was not severe in January 2013 in Spalding (Fig. 4e); however, in November 2013, phoma leaf spotting severity on Roxet was similar to that on Drakkar and Bilbao (score *c*. 2), with no significant differences between these three cultivars (*P *>* *0.05) and it was less on Excel (score 1.7) and Adriana (score 1.3).

### Effects of cropping season, site and cultivar on severity of stem base cankers and upper stem lesions

ANOVA of stem base canker or upper stem lesion severities on five cultivars in experimental plots at Banbury, Cowlinge, Harpenden, Rothwell and Spalding in the 2010/11, 2011/12, 2012/13 and 2013/14 cropping seasons showed that stem base canker or upper stem lesion severities differed significantly (*P *<* *0.01) between cropping seasons and between cultivars and sites.

**Figure 5:**
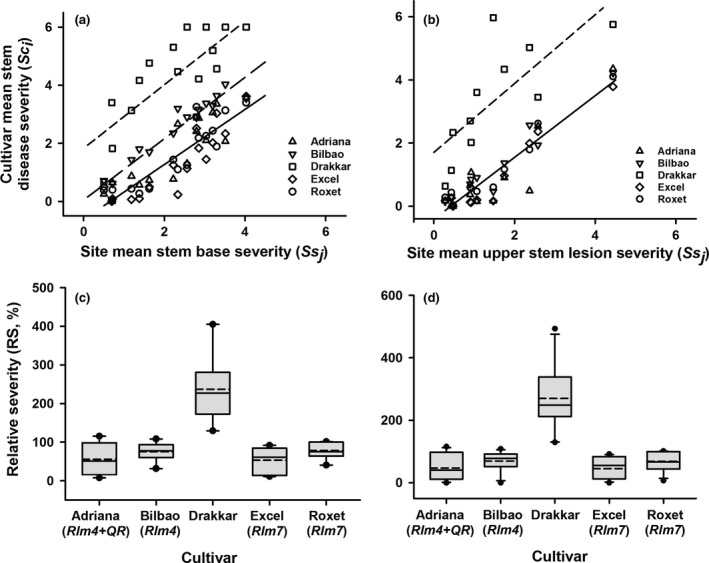
Relationship between mean stem base canker severity (a) or upper stem lesion severity (b) on each cultivar (*Sc*_*i*_) and mean stem base canker severity (a) or upper stem lesion severity (b) at each site (*Ss*_*j*_) over four cropping seasons (2010/11, 2011/12, 2012/13 and 2013/14). (a) Analysis of position and parallelism for stem base canker severity showed that these data best fitted three non‐parallel linear lines, accounting for 84.7% of the variation (74 d.f.). There was one line (*Sc*_*i*_ **= 1.10*Ss*_*j*_ **+ 1.83) for cultivar Drakkar (– – –), one line (*Sc*_*i*_ **= 1.06*Ss*_*j*_ **+ 0.05) for cultivar Bilbao (– – –) and one line (*Sc*_*i*_ **= 0.95*Ss*_*j *_− 0.63) for the cultivars Adriana, Roxet and Excel (^___^). (b) Analysis of position and parallelism for upper stem lesion severity showed that these data best fitted two non‐parallel linear lines, accounting for 84.2% of the variation (51 d.f.). There was one line (*Sc*_*i*_ **= 1.10 *Ss*_*j*_ **+ 1.71) for cultivar Drakkar (– – –) and one line (*Sc*_*i*_ **= 0.98 *Ss*_*j*_ **− 0.41) for the cultivars Adriana, Bilbao, Roxet and Excel (^___^). (c, d) Box‐plots showing the distribution in relative severity (RS) of stem base canker (c) or upper stem lesion (d) for each cultivar. The RS was calculated as a ratio and expressed as a percentage, $\text{RS}(\%)=\frac{Sc_{i}}{Ss_{j}}\times 100$ , where *Sc*_*i*_ is the mean disease severity for each cultivar *i* (Drakkar, Excel, Roxet, Adriana or Bilbao) and *Ss*_*j*_ is the mean disease severity at each site *j* (Banbury, Cowlinge, Harpenden, Rothwell or Spalding). Each box‐plot shows the mean (– – –) and the median (^___^) percentage RS. The lower and upper boundaries of the boxes indicate percentage RS for the 25th and 75th percentiles while whisker bars above and below each box indicate percentage RS for the 5th and 95th percentiles. Black dots below and above each box‐plot represent the minimum and maximum values, respectively.

Mean stem base canker or upper stem lesion severity for each cultivar was plotted against mean severity at each site and the relationships were linear (Fig. 5a,b). When an analysis of position and parallelism was done, three distinct groups identified for stem base canker severity were fitted best by three non‐parallel lines, accounting for 84.7% of the variation (74 d.f.; Fig. 5a) and two distinct groups were identified for upper stem lesion severity fitted best by two non‐parallel lines, accounting for 84.2% of the variation (51 d.f.; Fig. 5b). Thus, most severe stem base canker symptoms were on Drakkar (fitted by one line: *Sc*_*i*_ = 1.10*Ss*_*j*_ **+ 1.83), there was an intermediate group with Bilbao (fitted by one line: *Sc*_*i *_= 1.06*Ss*_*j *_+ 0.05) and Adriana, Roxet and Excel had the least severe stem base canker symptoms (fitted by one line: *Sc*_*i *_= 0.95*Ss*_*j*_ – 0.63; Fig. 5a). Most severe upper stem lesion symptoms were on Drakkar (fitted by one line: *Sc*_*i*_ = 1.10*Ss*_*j*_ + 1.71) and least severe were on Bilbao, Adriana, Roxet and Excel (fitted by one line: *Sc*_*i*_ = 0.98*Ss*_*j*_ – 0.41; Fig. 5b).

A statistical analysis of the distribution in the relative stem base canker or upper stem lesion severity on each cultivar (Fig. 5c,d) showed that the cultivars were grouped in the same way.

### Phoma stem canker severity over time

**Figure 6:**
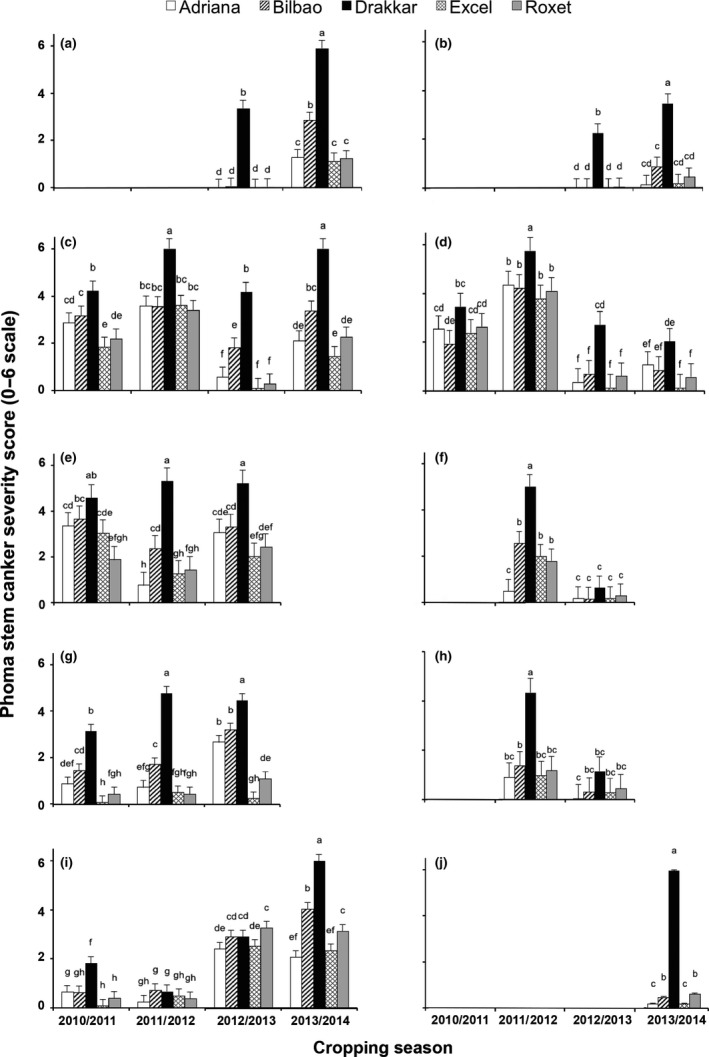
Severity of stem base canker (a, c, e, g, i) and upper stem lesions (b, d, f, h, j) on stems of oilseed rape cultivars Adriana [*Rlm4 *+* * quantitative resistance (QR)], Bilbao (*Rlm4*), Drakkar (no known *Rlm* gene), Excel and Roxet (both with *Rlm7*) in field experiments at Banbury (a, b), Cowlinge (c, d), Harpenden (e, f), Rothwell (g, h) and Spalding (i, j) in June/July before harvest in 2011, 2012, 2013 and 2014. Symptoms were considered to be upper stem lesions if they were observed >10 cm above the root crown and basal cankers if they were at the root crown or between the root crown and 10 cm above it. Stem base canker and upper stem lesion severity are expressed on a 0–6 scale (0: no disease observable; 1: 1–5% area of the stem cross‐section necrotic; 2: 6–25% area of the stem necrotic; 3: 26–50% area of the stem necrotic; 4: 51–75% area of the stem necrotic; 5: 76–100% area of the stem necrotic; 6: stem completely necrotic, dry/broken). The mean score was calculated from scores on 10 plants from each of the three replicate plots. Mean scores designated by the same letter were not significantly different (*P *> 0.05).

At Banbury, severity of stem base cankers and upper stem lesions was greater on Drakkar, Excel, Roxet, Adriana and Bilbao in 2014 than in 2013 (Fig. 6 a,b). At Cowlinge, the most severe stem base canker severity was observed on the susceptible cultivar Drakkar in July 2012 and in June 2014 (Fig. 6c). In July 2012, the stem base canker severity score was similar (*c*. 3.5) on Bilbao, Adriana, Roxet and Excel (*P *>* *0.05; Fig. 6c) and in June 2014 it was similar (*c*. 2.2) on Adriana and Roxet (*P *>* *0.05). The stem base canker severity score was >2 on the *Rlm7* cultivars at Harpenden in June 2011 and July 2013 (Fig. 6e). However, at Rothwell, stem base canker was generally not severe on the *Rlm7* cultivars in 2011, 2012 and 2013 (Fig. 6g). At Spalding, the stem base canker severity score was >2 on the *Rlm7* cultivars in July 2013 and in June 2014 (Fig. 6i).

Severe upper stem lesions were observed in July 2012 at Cowlinge, Harpenden and Rothwell with no statistically significant differences (*P *>* *0.05) between the four cultivars Adriana, Bilbao, Roxet and Excel, except on Adriana at Harpenden, on which they were less severe than on the other cultivars (Fig. 6d,f,h). Severe upper stem lesions were also observed on Drakkar at Spalding in June 2014 (Fig. 6j) and, at that time, scores of *c*. 0.4 were observed on the *Rlm7* cultivars.

### Detection of virulent allele frequencies in the *L. maculans* populations

**Figure 7:**
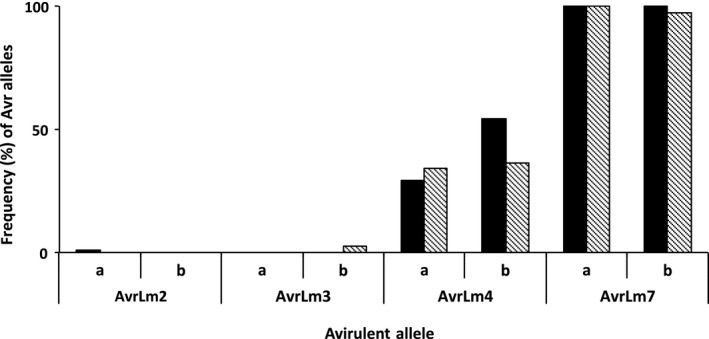
Frequencies (%) of the avirulent alleles *AvrLm2, AvrLm3, AvrLm4* and *AvrLm7* in populations of *Leptosphaeria maculans*. The isolates were obtained from leaves of the cultivars with the resistance gene *Rlm7* (Roxet and Excel; pattern columns) and the cultivar susceptible to *L. maculans* (Drakkar; black columns) in the winter of the (a) 2011/12 and (b) 2012/13 cropping seasons. *Leptosphaeria maculans* isolates obtained from two commercially available *Rlm7* cultivars (ExPower and Extrovert) from commercial crops in the 2011/12 cropping season and from one cultivar carrying the *Rlm7* gene included in a breeding programme by the company LS Plant Breeding (E1125) in the 2012/13 cropping season are also included.

All the tested isolates obtained from Drakkar in the 2011/12 (91 isolates) and 2012/13 (22 isolates) cropping seasons were virulent against *Rlm2* and *Rlm3* (i.e. *avrLm2* and *avrLm3* alleles) and avirulent against *Rlm7* (i.e. *AvrLm7* allele). Furthermore, 30% of the isolates obtained in the 2011/12 cropping season and *c*. 55% of the isolates obtained in the 2012/13 cropping season were avirulent against *Rlm4* (i.e. *AvrLm4* allele) (Fig. 7).

**Figure 8:**
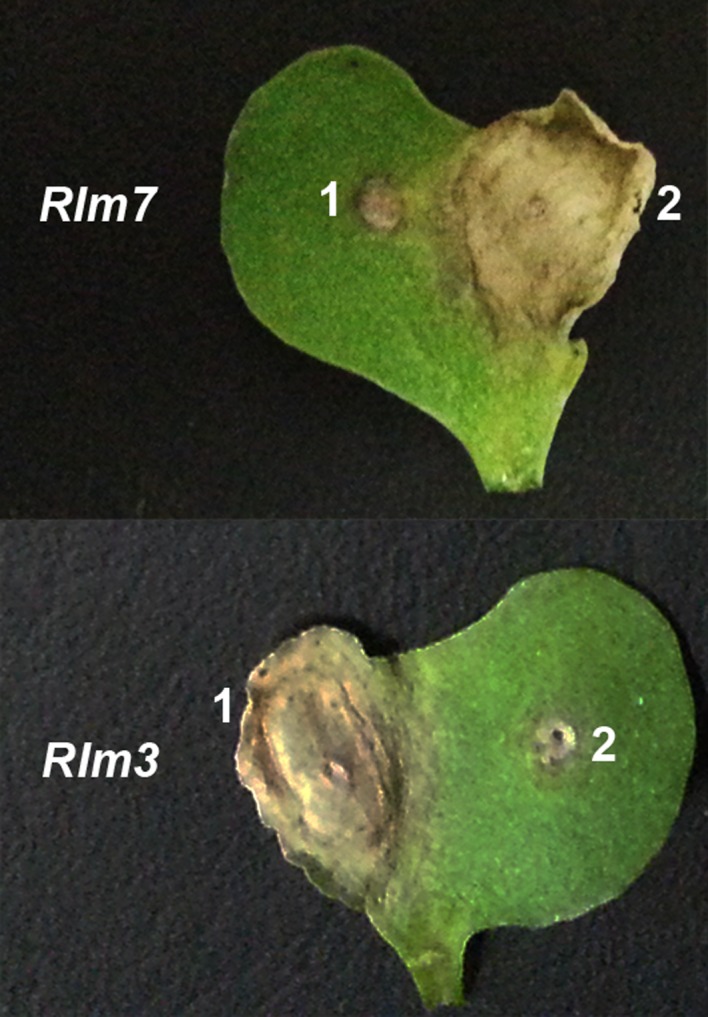
Lesions at 17 days post‐inoculation on representative cotyledons of the lines carrying the *Rlm7* (01‐23‐2‐1) or the *Rlm3* (02‐22‐2‐1) gene from the differential set used to determine *Avr* alleles in *Leptosphaeria maculans* isolates. Isolates were obtained from leaves of the cultivars carrying *Rlm7*, Excel and Roxet, in the winter of the 2012/13 cropping season. Isolate 1 was avirulent against *Rlm7* and virulent against *Rlm3* whereas isolate 2 was virulent against *Rlm7* and avirulent against *Rlm3*.

All the tested isolates obtained from the *Rlm7* cultivars (30 in the 2011/12 cropping season and 144 in the 2012/13 cropping season) were virulent against *Rlm2* (*avrLm2*). Moreover, all isolates obtained in the 2011/12 cropping season were virulent against *Rlm3* (*avrLm3*) and avirulent against *Rlm7* (*AvrLm7*). However, isolates that were virulent (3%) against *Rlm7* were detected in 2012/13 and they were avirulent against *Rlm3* (Fig. 8). These isolates had been obtained from Oadby Lodge Farm, Cowlinge and Bainton. About 35% of the isolates obtained from the *Rlm7* cultivars in the 2011/12 or in the 2012/13 cropping season were virulent against *Rlm4*.

### Phenotype and molecular events for the virulent *avrLm7 L. maculans* isolates

Two of the three virulent *avrLm7 L. maculans* isolates detected had been obtained from lesions with dark margins and one of them had been obtained from a grey lesion without a dark margin (Fig. S3a). No PCR product was amplified by either the Ext‐F/Ext‐R, Ext‐F3/Ext‐R or the Int‐F/Int‐R primer sets (Daverdin *et al*., 2012) in two *L. maculans* isolates that showed a virulent phenotype in the cotyledon test on the *Rlm7* line 01 (Fig. S3b); this indicated a deletion of the *AvrLm4‐7* gene. However, the *AvrLm4‐7* gene was amplified in one of the virulent isolates (virulent following the cotyledon test on the *Rlm7* line 01.23.2.1) using the same primer sets. The purified PCR product was sequenced and compared to the sequence of the reference avirulent allele (*AvrLm4‐7*; GenBank: AM998638.1; Fig. S3c). This revealed 20 point mutations affecting 11 triplets of nucleotides that resulted in 11 changes in the amino acids of the subsequent protein (Fig. S3d; Table S2a). Of the 11 affected codons, four had a single nucleotide change; four had two nucleotide changes and the remaining three had all nucleotides altered. The three codons that had all the three nucleotides altered were found before the 3′ UTR region. The most frequent nucleotide substitution was G>T (occurred four times), followed by A>G or C>T (both occurred three times; Table S2b). There was a predominance of GC to AT mutations (70%), that are associated with RIP mutations (Idnurm & Howlett, 2003).

### Evidence of the presence of QR and the *Rlm7* gene in Excel and Roxet

**Figure 9:**
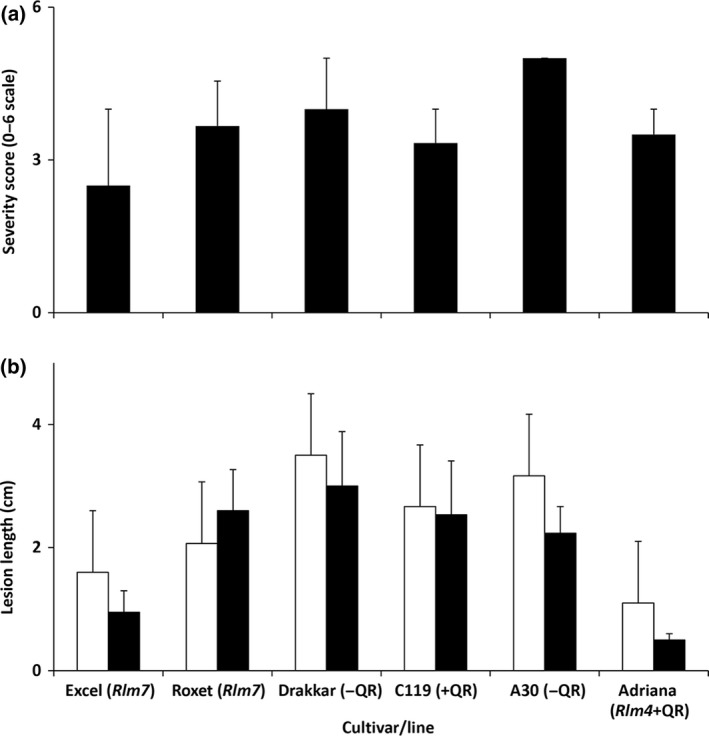
Development of phoma stem canker symptoms on oilseed rape cultivars/lines in controlled environment experiments to assess quantitative resistance (QR). Stem canker severity (a) on a 0–6 scale and lesion length (b) externally (black column) and internally (white column; after cutting the stem vertically at the point with external symptoms). Plants of oilseed rape cultivars Excel (with *Rlm7*), Roxet (with *Rlm7*), Drakkar (no known *Rlm* gene), doubled haploid (DH) line C119 (with QR), DH line A30 (susceptible) and the cultivar Adriana (with *Rlm4 *+** QR) were inoculated at the base of the petiole with a conidial suspension of *Leptosphaeria maculans* isolate H Rox 12.2.1 (*avrLm4, avrLm7*) and were incubated at 20 °C for 40 days. The error bars indicate the standard error of the difference between means (SEM; 4 d.f.).

Following inoculation of the petioles with a virulent *L. maculans* isolate (*avrLm4, avrLm7*), the oilseed rape cultivar Excel (with *Rlm7*) had the smallest phoma stem canker score (score 2.5) followed by that on line C119 (with QR; score 3.3) and cultivar Adriana (with *Rlm4 *+ QR; score 3.5; Fig. 9a). The greatest phoma stem canker score and the largest lesions were observed on the cultivar Drakkar and the line A30 (Fig. 9a,b), which are susceptible to *L. maculans* and do not have QR. The cultivar Excel had smaller lesion lengths internally and externally (*P *<* *0.05) and smaller severity score (*P *<* *0.10) than A30 or Drakkar. The cultivar Roxet had smaller lesion length externally (*P *<* *0.05) and smaller severity score (*P *<* *0.10) than A30 and Drakkar.

In cotyledon inoculation tests, all the isolates obtained from phoma leaf spots in the 2011/12 cropping season that were avirulent against *Rlm7* produced typical lesions of a susceptible phenotype on Drakkar (scores ranged from 4.9 to 5.8) and of a typical resistant phenotype on Excel (scores ranged from 1.1 to 1.9), on Roxet (scores ranged from 1.1 to 2.3) and on line 01.23.2.1 (scores ranged from 1.1 to 1.6; Table 1). Large grey lesions were produced on Drakkar, whereas small lesions surrounded by dark margins were produced on cotyledons of the cultivars and line 01.23.2.1 containing *Rlm7* (Fig. S4).

**Table 1:** Phoma leaf spot phenotype score (0–6 scale) of *Leptosphaeria maculans* isolates 17 days post‐inoculation on cotyledons of the susceptible cultivar Drakkar (no *Rlm* gene) and cultivars with the *Rlm7* gene (Excel, Roxet and line 01.23.2.1 of the differential set; Balesdent *et al*., 2002)

Isolate	Race^a^	Cultivar/line			
		Drakkar	Excel	Roxet	01.23.2.1
E Rox 11‐2	Av5‐6‐7 (8)	5.2	1.1	1.7	1.0
H Rox 11‐7	Av5‐6‐7 (8)	5.0	1.3	2.3	1.1
K Rox 11‐21	Av5‐6‐7 (8)	5.2	1.4	1.9	1.6
K Rox 11‐4	Av5‐6‐7 (8)	5.3	1.3	1.6	1.4
H Exc 11‐1	Av4‐5‐6‐7 (8)	5.6	1.4	1.6	1.3
J Exc 11‐2	Av1‐4‐5‐6‐7 (8)	5.8	1.6	1.7	1.6
J Exc 11‐7	Av1‐4‐5‐6‐7 (8)	4.9	1.1	1.1	1.4
J Exc dm 11‐2	Av5‐6‐7 (8)	4.9	1.9	1.8	1.3

a The race was determined following cotyledon inoculation to determine the frequencies of the corresponding *Avr* alleles in *L. maculans* isolates as described in the text. Av numbers indicate the loci for which the isolate is avirulent and numbers in parentheses indicate locus for which the allele has not been identified.

## Discussion

These results suggest that there have been changes in the effectiveness of the resistance gene *Rlm7* against *L. maculans*, cause of phoma stem canker, on winter oilseed rape in the UK. This is the first report of *L. maculans* populations virulent against the *Rlm7* gene in the UK, with 3% of the isolates obtained from *Rlm7* cultivars in the 2012/13 cropping season virulent at the *AvrLm7* locus. Previous studies analysing the UK *L. maculans* populations in 2002 did not detect any isolate that was virulent at the *AvrLm7* locus (Stachowiak *et al*., 2006), but since then *Rlm7* cultivars have been continuously included in the AHDB recommended lists. Breakdown of this important source of resistance against the phoma stem canker pathogen *L. maculans* would cause substantial losses to the oilseed rape breeding industry and to farmers.

The risk of losing the use of this important resistance gene is highlighted by the situation in France; currently, 50% of the French oilseed rape area is sown with *Rlm7* cultivars and 20% of the *L. maculans* populations is now virulent against this gene after 10 years of its use in commercial cultivars there (Balesdent *et al*., 2015). Although the resistance gene *Rlm7* has also been a source of resistance in UK commercial cultivars for more than 10 years (Clarke *et al*., 2011), it has not been used as widely in the UK as in France. The present study provides evidence that the proportion of the UK oilseed rape area sown with cultivars containing *Rlm7* had increased to 15% by 2013/14, which increases the risk that *Rlm7* will be rendered ineffective by changes in the *L. maculans* populations. The situation is similar in Germany, where more cultivars with *Rlm7* have been introduced and a low frequency of isolates virulent against *Rlm7* has been detected (Winter & Koopmann, 2016).

In the present investigation, 3% of *L. maculans* isolates were virulent against *Rlm7* at a time when cultivars with *Rlm7* represented *c*. 5% in the UK oilseed rape area. However, it is difficult to identify the exact geographical coverage of each cultivar grown in the UK, because the AHDB cultivar survey only considered cultivars that each made up >5% of the survey area. Cultivars making up <5% of the survey area in each cropping season represented 36–64% of the UK oilseed rape area (period 2009/10 to 2015/16) and the *Rlm7* gene might have also been present in those cultivars. Breeding companies estimate that the use of the *Rlm7* gene in commercial crops in the UK had increased to >20% of the oilseed rape area by 2016/17.

The effectiveness of the *Rlm7* gene for control of *L. maculans* disease epidemics has lasted longer than that of other resistance genes against *L. maculans* (Clarke *et al*., 2011). For example, *Rlm1* in France was rendered ineffective after 3 years of extensive commercial use of cultivars with this gene, by which time >80% of the *L. maculans* populations were virulent against *Rlm1* (Rouxel *et al*., 2003). However, the detection of isolates virulent against the *Rlm7* gene and the increase in phoma leaf spotting on *Rlm7* cultivars over the four UK cropping seasons examined (2011–2014) shows that *L. maculans* populations in the UK are becoming virulent against the *Rlm7* gene. Changes in the symptoms caused by *L. maculans* on *Rlm7* cultivars from small lesions with dark margins to larger lesions without dark margins also suggest that there are changes in the pathogen populations. However, relationships between *L. maculans* lesion phenotype and mechanisms of virulence at the *AvrLm7* locus could not be determined, although there was a decrease in the frequency of lesions with dark margins observed from the 2011/12 to the 2012/13 cropping season.

Surprisingly, even if *L. maculans* isolates were obtained from phoma leaf spot lesions on *Rlm7* cultivars (i.e. in the 2011/12 or 2013/14 cropping seasons), they were not identified as virulent at the *AvrLm7* locus according to the cotyledon test method (Balesdent *et al*., 2001). This phenomenon was also observed in field trials in France, which showed that isolates with the avirulent allele *AvrLm7* were able to produce phoma leaf spot symptoms on *Rlm7* cultivars (Pinochet *et al*., 2013).

Previous work in France, involving use of cultivars with *Rlm7* at the same field site in successive cropping seasons, has described a number of different molecular events that have led to virulence towards *Rlm7*, where complete deletion of the *AvrLm7* gene became more common in the second and third year of the study (Daverdin *et al*., 2012). In the first year of the study, RIP mutation was the most frequent event leading to virulence against *Rlm7*, but its frequency had decreased by the second year. In the present investigation, results suggest that complete deletion of the gene might have been the mutation event in two out of the three UK *L. maculans* isolates that were associated with the virulent phenotype. RIP mutations were associated with lack of recognition by *Rlm7* in the other virulent isolate examined. If predominance of gene deletion is associated with pathogen evolution to virulence at the *Rlm7* locus, the present study suggests that the UK *L. maculans* populations are evolving to evade recognition by *Rlm7*.

It is interesting that the *L. maculans* isolates that were virulent against *Rlm7* were found to be avirulent against *Rlm3*, even though the UK populations have been previously found to be 100% virulent against *Rlm3* (Stachowiak *et al*., 2006). This can be explained by recent findings about the interaction between *AvrLm4‐7* and *AvrLm3* in pathogen populations in France where *Rlm3*‐mediated resistance is ‘masked’ in the presence of *AvrLm7*, the allele avirulent against *Rlm7* (Plissonneau *et al*., 2016).

The cultivars Excel and Roxet are *Rlm7* cultivars that have been included in previous studies (Clarke *et al*., 2011; Daverdin *et al*., 2012; Larkan *et al*., 2016). In the present study, results confirmed the presence of the *Rlm7* genes in these cultivars; in addition, QR against *L. maculans* was detected in Excel and Roxet using the method of Huang *et al*. (2014b) for petiole inoculation with an *L. maculans* isolate virulent to *Rlm7*, suggesting that background QR played a role in limiting severe phoma stem canker in these two cultivars. This suggests that, as well as the role of *Rlm7* in these cultivars to prevent severe phoma leaf spotting at the beginning of the cropping season, QR had a significant contribution in controlling phoma stem canker development at the end of the cropping season. Classification according to the severity of basal phoma stem canker in experimental plots placed the cultivars Adriana, Excel and Roxet (each with *Rlm* gene + QR) in one group, and Bilbao (*Rlm4*, no QR) in a separate group, with more severe symptoms. This provides strong evidence that combination of an *Rlm* gene in a cultivar with background QR is better for controlling phoma stem canker than a cultivar with just an *Rlm* gene. Quantitative resistance has been thought to be expressed at later stages of crop development, not preventing host colonization but decreasing symptom severity and epidemic progress over time (Huang *et al*., 2009; Brun *et al*., 2010; Delourme *et al*., 2014). Therefore, a combination of *Rlm* gene resistance and QR in oilseed rape is a primary objective for the breeding industry and should be the basis for disease management strategies.

The observation that severe phoma leaf spotting on the cultivar susceptible to *L. maculans* (Drakkar) in autumn was associated with subsequent severe phoma stem canker at the end of the cropping season was consistent with previous studies (Sun *et al*., 2001). However, in the present work, there were cases where substantial phoma stem canker on cultivars with *Rlm* genes observed in summer was not associated with severe phoma leaf spotting by *L. maculans* in the previous autumn/winter (e.g. at Cowlinge and Spalding in 2012/13 and at Rothwell in 2011/12). The contribution of the closely related pathogen *L. biglobosa* to phoma stem canker development should also be considered. Even if *L. biglobosa* has been considered as less damaging and associated with upper stem lesions (West *et al*., 2001; Fitt *et al*., 2006a), this pathogen has been found to contribute to phoma stem canker development in field experiments in the UK (Huang *et al*., 2014a). Further investigation is required to examine the importance of this pathogen in the development of disease epidemics.

It is essential that the *Rlm7* gene is used wisely by the breeding and farming industries to enable its continued use for control of the disease. A combination of precise use of fungicides to control severe disease epidemics (Huang *et al*., 2011; Sewell *et al*., 2016) and the use of cultivars with effective *Rlm* genes decreases severity of epidemics and thus reduces the concentration of primary ascospore inoculum for starting epidemics in the following cropping season. It is also very important to use *Rlm* genes in cultivars with quantitative resistance (Brun *et al*., 2010) to maintain effectiveness of important *Rlm* genes, such as *Rlm7* (Hayward *et al*., 2012; Mundt, 2014). In the context of sustainable disease management, further strategies to reduce primary ascospore inoculum (i.e. crop rotation, cultural methods and separation of successive oilseed rape crops; Marcroft *et al*., 2012b) also need to be employed.

Strategies for guided deployment of cultivars in space and time (Gladders *et al*., 2006), depending on their complement of *Rlm* genes and on *L. maculans* populations at different locations (such as disease management strategies employed in Australia, https://www.grdc.com.au; France, http://www.terresinovia.fr and http://www.myvar.fr; and Canada, http://www.canolacouncil.org), should be considered as methods to enable prolonged use of important resistance genes against *L. maculans*, such as *Rlm7*, and effective control of phoma stem canker in the UK.

## Supporting information


**Figure S1.** Map with locations of sites of winter oilseed rape field experiments in England.

**Figure S2.** Percentage of the total oilseed rape area in England, Scotland and Wales planted to each oilseed rape cultivar (A–Q or ‘Others’) in the period covering the 2009/10 to 2015/16 cropping seasons.

**Figure S3.** Phenotypes and molecular events associated with the *avrLm7* (virulent) phenotype. (a) Phenotypes of lesions produced by *Leptosphaeria maculans* isolates containing *avrLm7* (I1 Exc 12‐8‐1, H Rox 12‐2‐1, E1125 12‐5‐2) before incubation for pycnidial production and pathogen isolation, after incubation, and following the cotyledon test on the line 01.23.2.1 containing *Rlm7*. (b) Results of PCR of *L. maculans* isolates that had shown a virulent phenotype in the cotyledon phenotype test on the *Rlm7* line 01.23.2.1. The *AvrLm4‐7* gene was amplified using external or internal primer sets (Daverdin *et al*., 2012). (c) Nucleotide sequence alignment, using clustal v. 2.1, of the *AvrLm4‐7* gene amplified in a *L. maculans* isolate that showed a virulent phenotype against *Rlm7*. The sequence of the reference avirulent allele (*AvrLm4‐7*; GenBank: AM998638.1) was used to compare the nucleotide sequence following sequencing of purified PCR product of the virulent *L. maculans* isolate I1 Exc 12‐8‐1 using the Ext‐F/Ext‐R primer set. (d) Amino acid sequence alignment, using clustal v. 2.1, of the AvrLm4‐7 protein in a *L. maculans* isolate that showed a virulent phenotype against *Rlm7*. EMBOSS transeq was used to translate the nucleotide sequence to amino acid sequence of the sequenced PCR product using the Ext‐F/Ext‐R primer set of the virulent *L. maculans* isolate I1 Exc 12‐8‐1. This was compared to the amino acid sequence of the reference avirulent allele (AvrLm4‐7; protein_id=CAQ53119.1) of the *AvrLm4‐7* gene.

**Figure S4.** Phenotypes of *Leptosphaeria maculans* isolates carrying *AvrLm7*, 17 days post‐inoculation on cotyledons of the susceptible Drakkar (no *Rlm* gene) and cultivars with the *Rlm7* gene [Excel, Roxet and line 01.23.2.1 of the differential set (Balesdent *et al*., 2002)].

**Table S1.** Equations used to analyse the relationship between mean severity of phoma leaf spotting or stem canker for each cultivar (*Sc*_*i*_) and mean severity of phoma leaf spotting or stem canker at each site (*Ss*_*j*_).

**Table S2.** Total nucleotide changes and the resulting amino acid changes in the *AvrLm4‐7* gene and protein in a *Leptosphaeria maculans* isolate that showed a virulent phenotype. (a) Repeat‐induced point mutations and the resulting amino acid changes; (b) nucleotide changes and the frequency with which they occurred in the virulent *L. maculans* isolate I1 Exc 12‐8‐1.
