# Supplementary material for: Effectiveness of Rlm7 resistance against Leptosphaeria maculans (phoma stem canker) in UK winter oilseed rape cultivars
Source: Plant Pathol. 2018 Mar 23;67(6):1339–53. doi: 10.1111/ppa.12845 (PMC6108410; doi:10.1111/ppa.12845)

**Supporting** **Figure 1**

Map with locations of sites of winter oilseed rape field experiments in England. 1: Bainton, 2: Banbury, 3: Cowlinge, 4: Harpenden, 5: Harper Adams, 6: Horncastle, 7: Morley, 8: Oadby Lodge Farm, 9: Rothwell, 10: Spalding, 11: Stockbridge


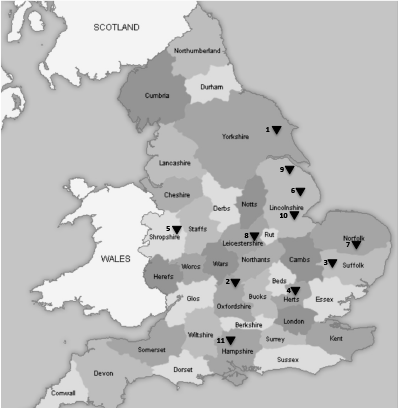

Supplement: Supplementary file 1 — Figure S1. Map with locations of sites of winter oilseed rape field experiments in England. [file PPA-67-1339-s001.docx]
