# Supplementary material for: Effectiveness of Rlm7 resistance against Leptosphaeria maculans (phoma stem canker) in UK winter oilseed rape cultivars
Source: Plant Pathol. 2018 Mar 23;67(6):1339–53. doi: 10.1111/ppa.12845 (PMC6108410; doi:10.1111/ppa.12845)
Supplement: Supplementary file 2 — Figure S2. Percentage of the total oilseed rape area in England, Scotland and Wales planted to each oilseed rape cultivar (A–Q or ‘Others’) in the period covering the 2009/10 to 2015/16 cropping seasons. [file PPA-67-1339-s002.docx]

**Supporting** **Figure 2**

Percentage of the total oilseed rape area in England, Scotland and Wales planted to each oilseed rape cultivar (A-Q or “Others”) in the period covering the 2009/10 – 2015/16 cropping seasons. The graph was generated by using the oilseed rape cultivar survey data collected by AHDB (representing c. 10-13% of the total UK oilseed rape area for each of the cropping seasons) (www.ahdb.org.uk). * Other cultivars each individually accounted for less than 5% of the area surveyed. Red circles indicate the cultivars with the *Rlm7* gene against *L. maculans* based on experiments done by INRA, France, by inoculating seedlings with isolates with different combinations of *Avr* alleles (differential set of *L. maculans* isolates) or information communicated about the presence/absence of the *Rlm7* gene by the specific breeding companies.
