# Supplementary material for: Effectiveness of Rlm7 resistance against Leptosphaeria maculans (phoma stem canker) in UK winter oilseed rape cultivars
Source: Plant Pathol. 2018 Mar 23;67(6):1339–53. doi: 10.1111/ppa.12845 (PMC6108410; doi:10.1111/ppa.12845)
Supplement: Supplementary file 3 — Figure S3. Phenotypes and molecular events associated with the avrLm7 (virulent) phenotype. (a) Phenotypes of lesions produced by Leptosphaeria maculans isolates containing avrLm7 (I1 Exc 12‐8‐1, H Rox 12‐2‐1, E1125 12‐5‐2) before incubation for pycnidial production and pathogen isolation, after incubation, and following the cotyledon test on the line 01.23.2.1 containing Rlm7. (b) Results of PCR of L. maculans isolates that had shown a virulent phenotype in the cotyledon phenotype test on the Rlm7 line 01.23.2.1. The AvrLm4‐7 gene was amplified using external or internal primer sets (Daverdin et al., 2012). (c) Nucleotide sequence alignment, using clustal v. 2.1, of the AvrLm4‐7 gene amplified in a L. maculans isolate that showed a virulent phenotype against Rlm7. The sequence of the reference avirulent allele (AvrLm4‐7; GenBank: AM998638.1) was used to compare the nucleotide sequence following sequencing of purified PCR product of the virulent L. maculans isolate I1 Exc 12‐8‐1 using the Ext‐F/Ext‐R primer set. (d) Amino acid sequence alignment, using clustal v. 2.1, of the AvrLm4‐7 protein in a L. maculans isolate that showed a virulent phenotype against Rlm7. EMBOSS transeq was used to translate the nucleotide sequence to amino acid sequence of the sequenced PCR product using the Ext‐F/Ext‐R primer set of the virulent L. maculans isolate I1 Exc 12‐8‐1. This was compared to the amino acid sequence of the reference avirulent allele (AvrLm4‐7; protein_id=CAQ53119.1) of the AvrLm4‐7 gene. [file PPA-67-1339-s003.docx]

**Supporting** **Figure 3**

Phenotypes and molecular events associated with the *avrLm7* (virulent) phenotype.

**a.** Phenotypes of lesions produced by *avrLm7* *L. maculans* isolates (I1 Exc 12-8-1, H Rox 12-2-1, E1125 12-5-2) before incubation for pycnidial production and pathogen isolation, after incubation and following the cotyledon test on the *Rlm7* line (01.23.2.1) of the differential set of brassica cultivars/lines (scale bars: 0.5 cm).

**b.** *Leptosphaeria maculans* isolates that showed a virulent phenotype in the cotyledon phenotype test on the *Rlm7* line 01.23.2.1 were used to determine the molecular events at the *AvrLm7* locus. The *AvrLm4-7* gene was amplified using external or internal primer sets (Daverdin *et al*. 2012). DirectLoad™ 1 kb DNA Ladder ready-to-use marker for DNA electrophoresis or PCR 100 bp Low Ladder for electrophoresis of PCR fragments (Sigma-Aldrich, UK) are indicated.

**c.** Nucleotide sequence alignment of the *AvrLm4-7* gene amplified in a *Leptosphaeria maculans* isolate that showed a virulent phenotype using CLUSTAL 2.1. The sequence of the reference avirulent allele (*AvrLm4-7*; GenBank: AM998638.1) was used to compare the nucleotide sequence following sequencing (SUPREMERUN, Sanger sequencing, GATC, Germany) of purified PCR product of the virulent *L. maculans* isolate I1 Exc 12-8-1 using the Ext-F/Ext-R primer set. The sequences were independently read two or three times with the primers.

**d.** Amino acid sequence alignment of the AvrLm4-7 protein in a *Leptosphaeria maculans* isolate that showed a virulent phenotype using CLUSTAL 2.1. EMBOSS Transeq tool was used to translate the nucleotide sequence to amino acid sequence of the sequenced (SUPREMERUN, Sanger sequencing, GATC, Germany) purified PCR product using the Ext-F/Ext-R primer set of the virulent *L. maculans* isolate I1 Exc 12-8-1. This was compared to the amino acid sequence of the reference avirulent allele (AvrLm4-7; protein_id=CAQ53119.1) of the *AvrLm4-7* gene.

**a.**

**b.**


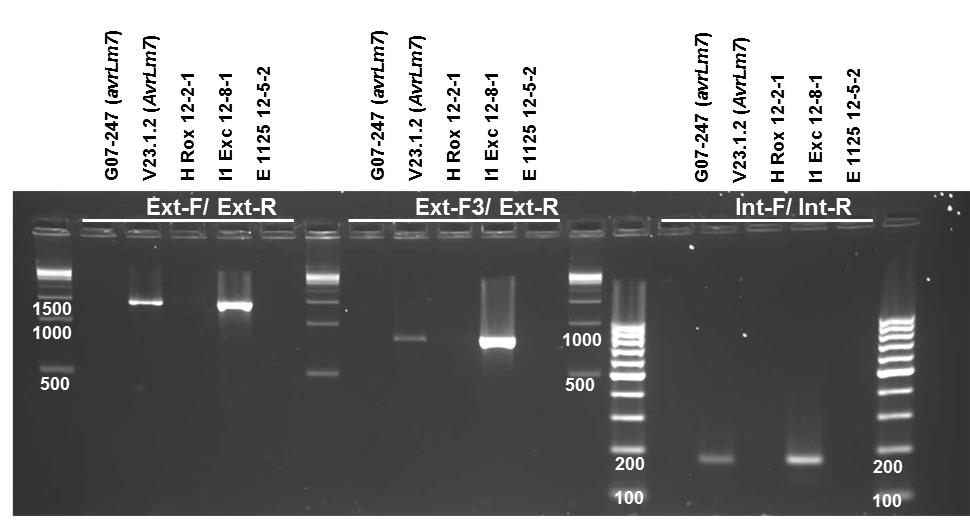


**Ladder**

**Ladder**

**Ladder**

**Ladder**

**Ladder**

**c.**


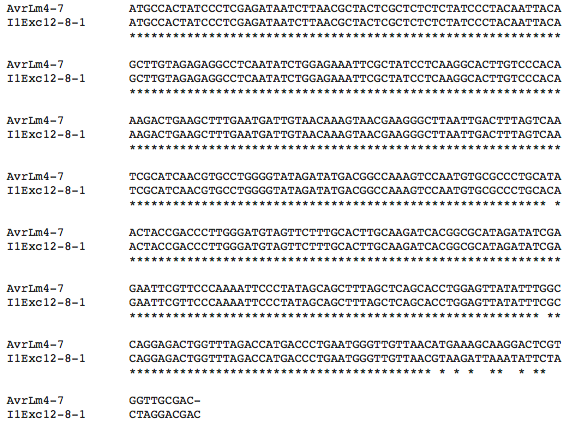


An * (asterisk) indicates positions which have a single, fully conserved residue.

Absence of an asterisk indicates positions which have a single change in the residue.

**d.**


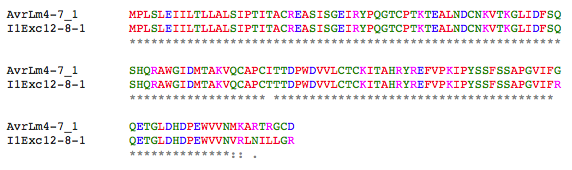


An * (asterisk) indicates positions which have a single, fully conserved residue.

A : (colon) indicates conservation between groups of strongly similar properties - scoring > 0.5 in the Gonnet PAM 250 matrix.

A . (period) indicates conservation between groups of weakly similar properties - scoring =< 0.5 in the Gonnet PAM 250 matrix.

Absence of a symbol indicates no conservation.

The residues are coloured according to their physicochemical properties using CLUSTAL 2.1.
