# Supplementary material for: Effectiveness of Rlm7 resistance against Leptosphaeria maculans (phoma stem canker) in UK winter oilseed rape cultivars
Source: Plant Pathol. 2018 Mar 23;67(6):1339–53. doi: 10.1111/ppa.12845 (PMC6108410; doi:10.1111/ppa.12845)

**Supporting Figure 4**

Phenotype of *L. maculans* isolates 17 dpi on cotyledons of the susceptible Drakkar (no *Rlm* gene) and cultivars with the *Rlm7* gene (Excel, Roxet and line 01.23.2.1 of the differential set (Balesdent *et al.*, 2002)).


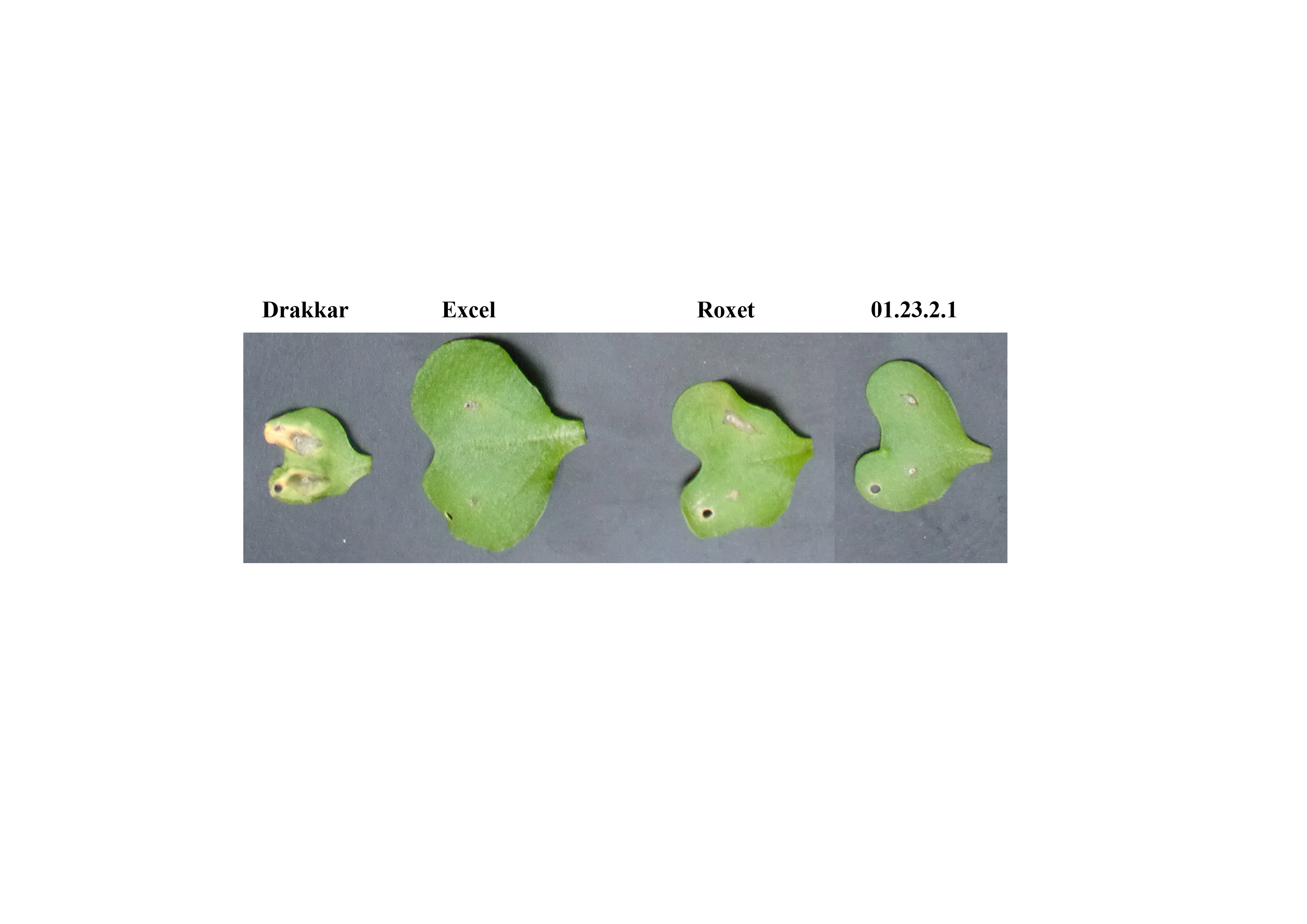

Supplement: Supplementary file 4 — Figure S4. Phenotypes of Leptosphaeria maculans isolates carrying AvrLm7, 17 days post‐inoculation on cotyledons of the susceptible Drakkar (no Rlm gene) and cultivars with the Rlm7 gene [Excel, Roxet and line 01.23.2.1 of the differential set (Balesdent et al., 2002)]. [file PPA-67-1339-s004.docx]
