# Supplementary material for: Effectiveness of Rlm7 resistance against Leptosphaeria maculans (phoma stem canker) in UK winter oilseed rape cultivars
Source: Plant Pathol. 2018 Mar 23;67(6):1339–53. doi: 10.1111/ppa.12845 (PMC6108410; doi:10.1111/ppa.12845)
Supplement: Supplementary file 5 — Table S1. Equations used to analyse the relationship between mean severity of phoma leaf spotting or stem canker for each cultivar (Sc i) and mean severity of phoma leaf spotting or stem canker at each site (Ss j). [file PPA-67-1339-s005.docx]

Supporting Table 1

Equations used to analyse the relationship between mean severity of phoma leaf spotting or stem canker for each cultivar (${Sc}_{i}$) and mean severity of phoma leaf spotting or stem canker at each site (${Ss}_{j}$).

| **Disease score** | **Equations** |
| --- | --- |
| Mean disease severity^a^ for each cultivar | ${Sc}_{i}=\frac{S_{r1}+S_{r2}+S_{r3}}{3}$ |
| Mean disease severity at each site | ${Ss}_{j}=\frac{{Sc}_{1}+{Sc}_{2}+\ldots+ {Sc}_{n}}{n}$ |
| Relative disease severity | $RS (\%)=\frac{{Sc}_{i}}{{Ss}_{j}} x 100$ |
| where $S_{r}$ is the disease severity for each of the three replicate plots for each cultivar, *i* is each of the cultivars (Adriana, Bilbao, Drakkar, Excel or Roxet) and *j* is each of the sites (Banbury, Cowlinge, Harpenden, Rothwell or Spalding).  where $S_{r}$ is the disease severity for each of the three replicate plots for each cultivar, *i* is each of the cultivars (Adriana, Bilbao, Drakkar, Excel or Roxet) and *j* is each of the sites (Banbury, Cowlinge, Harpenden, Rothwell or Spalding). | |

^a^ Phoma leaf spotting severity was on a 0-3 scale; stem base canker or upper stem lesion severity was on a 0-6 scale (modified from that of Lô-Pelzer *et al*., 2009).
