# Supplementary material for: Effectiveness of Rlm7 resistance against Leptosphaeria maculans (phoma stem canker) in UK winter oilseed rape cultivars
Source: Plant Pathol. 2018 Mar 23;67(6):1339–53. doi: 10.1111/ppa.12845 (PMC6108410; doi:10.1111/ppa.12845)
Supplement: Supplementary file 6 — Table S2. Total nucleotide changes and the resulting amino acid changes in the AvrLm4‐7 gene and protein in a Leptosphaeria maculans isolate that showed a virulent phenotype. (a) Repeat‐induced point mutations and the resulting amino acid changes; (b) nucleotide changes and the frequency with which they occurred in the virulent L. maculans isolate I1 Exc 12‐8‐1. [file PPA-67-1339-s006.docx]

Supporting Table 2

: Total nucleotide changes and the resulting amino acid changes in the *AvrLm4-7* gene and protein in a *Leptosphaeria maculans* isolate that showed a virulent phenotype.

(**a**) Repeat Induced Point mutations and the resulting amino acid changes; (**b**) nucleotide changes and the frequency with which they occurred in the virulent *L. maculans* isolate I1 Exc 12-8-1.

**a
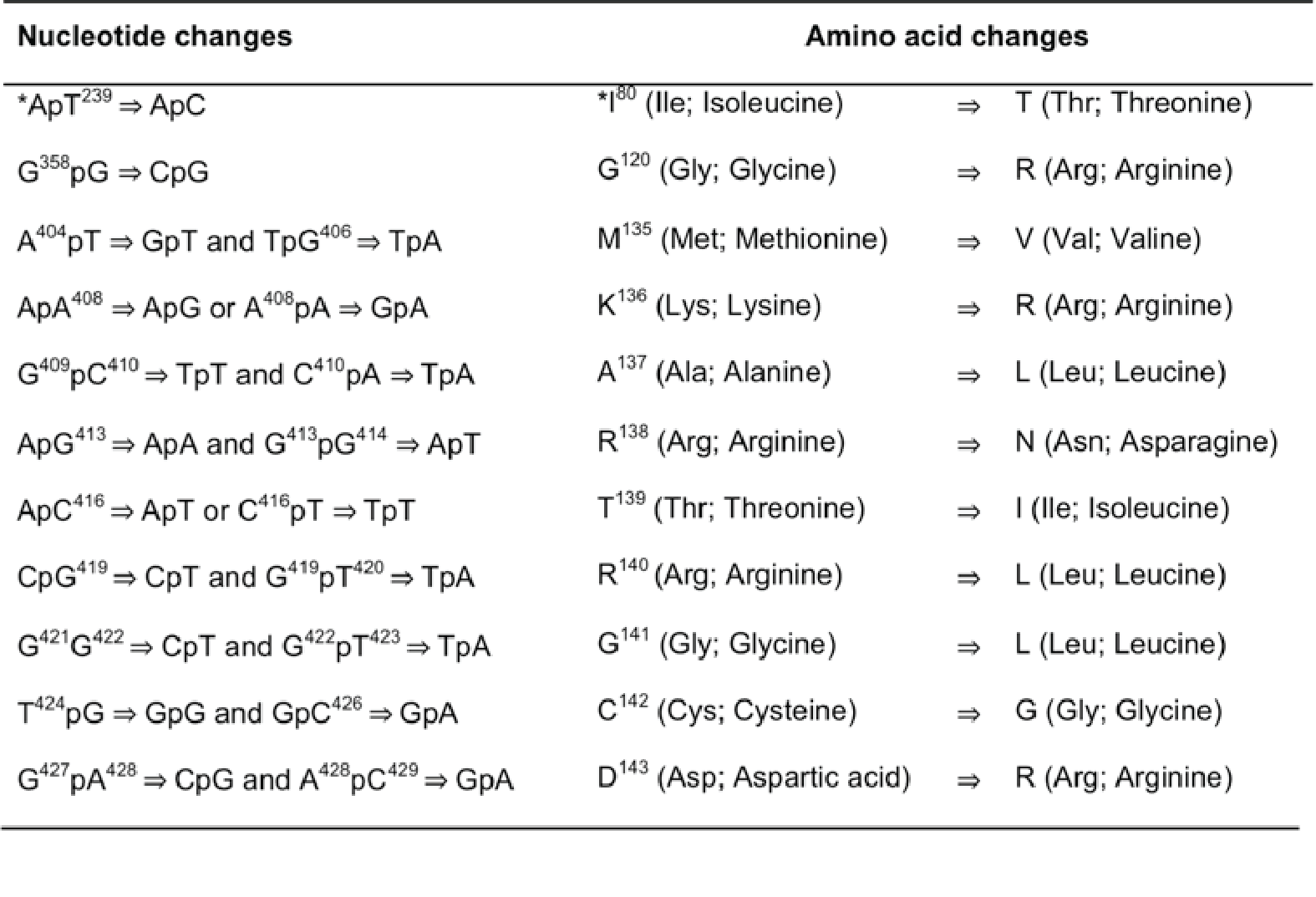
*** Numbers indicate the position in the gene (nucleotide) or protein (amino acid), respectively, where change occurred.

**b**

| **Nucleotide substitution** | **Frequency** |
| --- | --- |
| A⇒G | 3 |
| G⇒A | 2 |
| G⇒T | 4 |
| C⇒T | 3 |
| T⇒A | 2 |
| G⇒C | 2 |
| T⇒G | 1 |
| C⇒A | 2 |
